# Shedding light on dark adaptation

**DOI:** 10.1042/BIO20200067

**Published:** 2020-10-09

**Authors:** Ellen Weiss

**Affiliations:** The University of North Carolina at Chapel Hill, USA

## Abstract

The retina is famous for its ability to operate under a broad range of light intensities. This is partly due to the presence of two types of photoreceptor cells, rods and cones. Rods are used mostly for dim light vision, and cones are used for bright light and colour vision. These cells are also able to adapt to a broad range of light intensities using light- and dark-adaptation mechanisms. Dark adaptation is used by the vertebrate retina to increase its visual sensitivity when moving from a brightly lit environment to a dark environment. The brighter the surrounding light, the longer it takes for the retina to adapt to the dark. Most retina biologists have studied dark adaptation by exposing animals to a 90% bleach, meaning that 90% of the light-sensing proteins in these photoreceptor cells have been activated, followed by transfer of these animals to a dark room and analysis of their light sensitivity using electrophysiological methods. In this report, we introduce the basic elements of the visual system and describe how the system might operate during dark adaptation. We also introduce a novel role for cAMP-mediated phosphorylation of G protein-coupled receptor kinase 1 (GRK1), a major kinase in visual signalling.

Approximately 600m years ago, organisms acquired the ability to sense light for identifying obstacles, the motion of predators and developing a circadian rhythm. Image sensing and true visual detection evolved during the Cambrian explosion, approximately 540m years ago; by 500m years ago, the complex multifaceted retina that vertebrates use today had developed, with its high sensitivity, ability to detect colour, form and motion, and its high visual acuity. In addition, the pupil regulates the amount of light that is focused by the lens onto the retina. Also, the musculature external to the eye allows it to view objects at different distances and angles. This amazing structure can detect changes in light levels that range over 10 orders of magnitude. As Richard Masland stated in a 2012 review in *Neuron*: “Charles Darwin famously wrote that the eye caused him to doubt that random selection could create the intricacies of nature. Fortunately, Darwin did not know the structure of the retina: if he had, his slowly gestating treatise on evolution might never have been published at all”.

## The structure of the vertebrate retina

**Figure 1.**
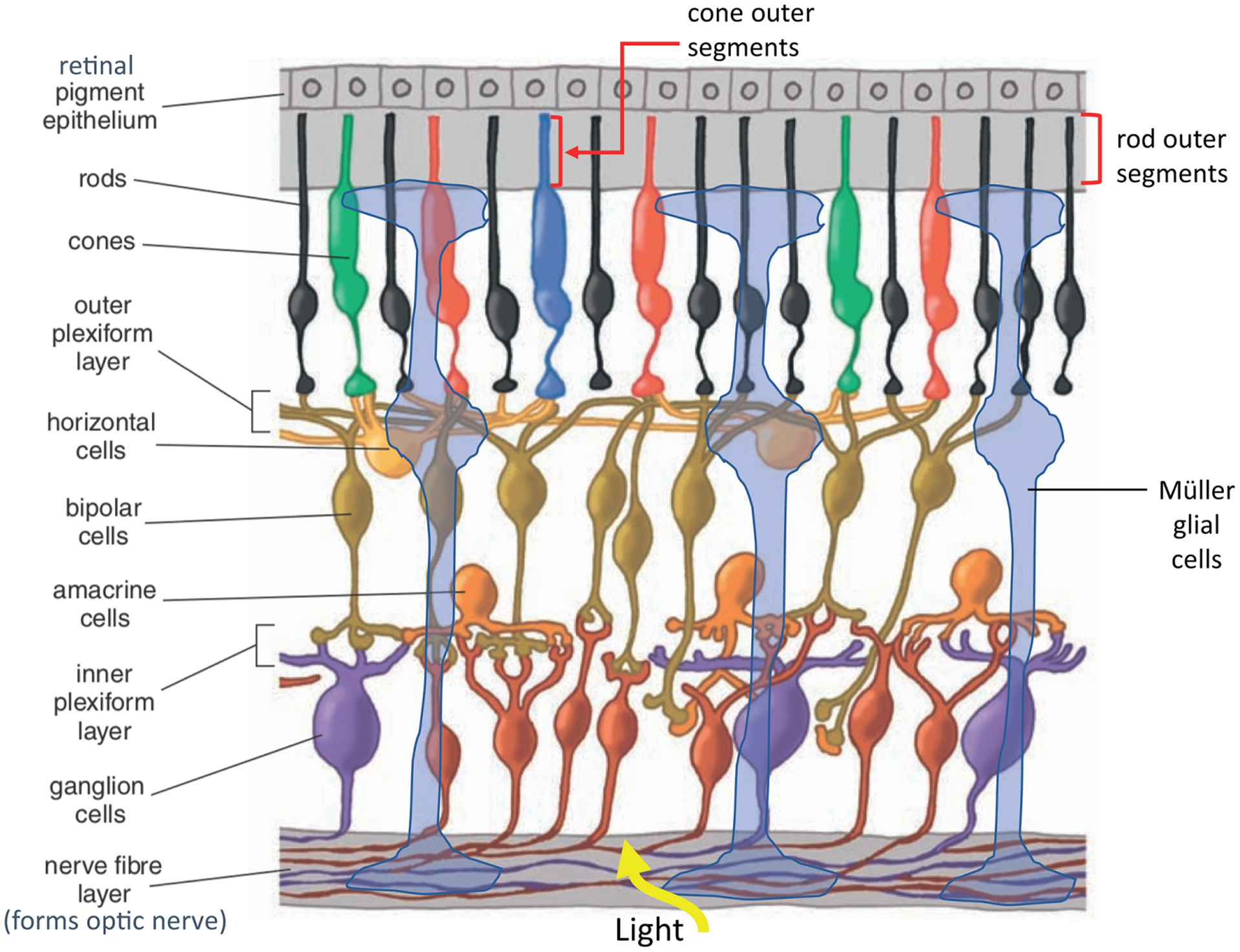
The cells in the vertebrate retina. See text for explanation of their functions. Modified from Kolb (2013) Amer. Scientist **91,** 28–35.

The vertebrate retina is made up of multiple cell types (Figure 1). Photoreceptors include rods, which are responsible for the detection of dim light, and cones, which function in bright light and are responsible for the ability to distinguish colours based on their unique spectral sensitivities. These cells each have a ciliary process, known as an outer segment, that consists of stacks of membranous discs where the proteins involved in light sensing and signalling are located. The rods and cones connect to bipolar cells. There are also neurons responsible for modifying visual signals, such as amacrine cells, which connect rod bipolar cells to cone bipolar cells, and horizontal cells, which mediate feedback inhibition to the photoreceptors. The cone bipolar cells connect to ganglion cells, which integrate the signals from the upstream neurons. The ganglion cell axons assemble to form the optic nerve for transmission of visual signals to the brain. Non-neuronal cells, the Müller glia, are responsible for maintenance of the retina and, in lower vertebrates, can revert to stem cells in order to redevelop all of the cell types in cases of injury. Efforts to replicate this process in the Müller glia of higher vertebrates, for therapy in patients with retinal degenerative diseases, is an active field of research. The Müller glia are also involved in retinoid synthesis necessary for rapid recovery of cone photoreceptors under constant bright light. The retinal pigment epithelium (RPE) is located at the periphery of the neural retina. These cells perform numerous housekeeping functions, including recycling of retinoids essential for light detection in rods and phagocytosis of some of the discs from the tips of the rod and cone outer segments. The rods and cones replace these discs with new discs each day at the base of each outer segment. An average RPE cell interacts with 20–30 rod outer segments and one cone outer segment.

## The vertebrate retina: response to moderate light intensities

**Figure 2.**
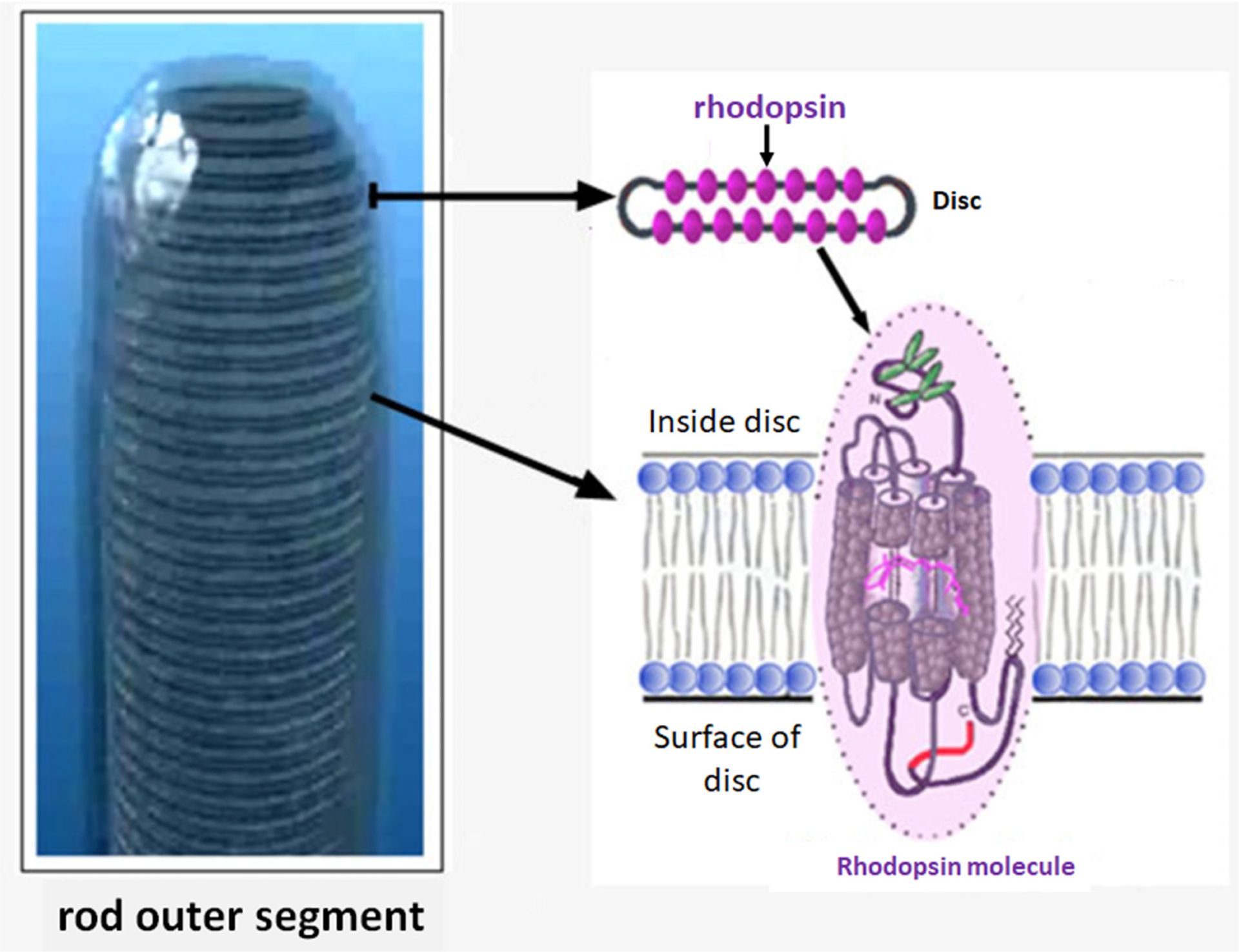
Structure of the rod outer segment with densely packed discs, each expressing thousands of rhodopsin molecules. Note that the stacked discs are contained within the plasma membrane of rod outer segment. Each stacked disc contains multiple rhodopsin molecules oriented with the carboxyl terminus facing the cytoplasm (surface of the disc) where GRK1, G_t1_ and arrestin are located, as well as other regulators of visual signalling. 11-*cis*-retinal is shown within the transmembrane domains of rhodopsin. Adapted from Webvision, webvision.med.utah.edu/. Noncommercial 4.0 International (CC BY-NC) Creative Commons license.

**Figure 3.**
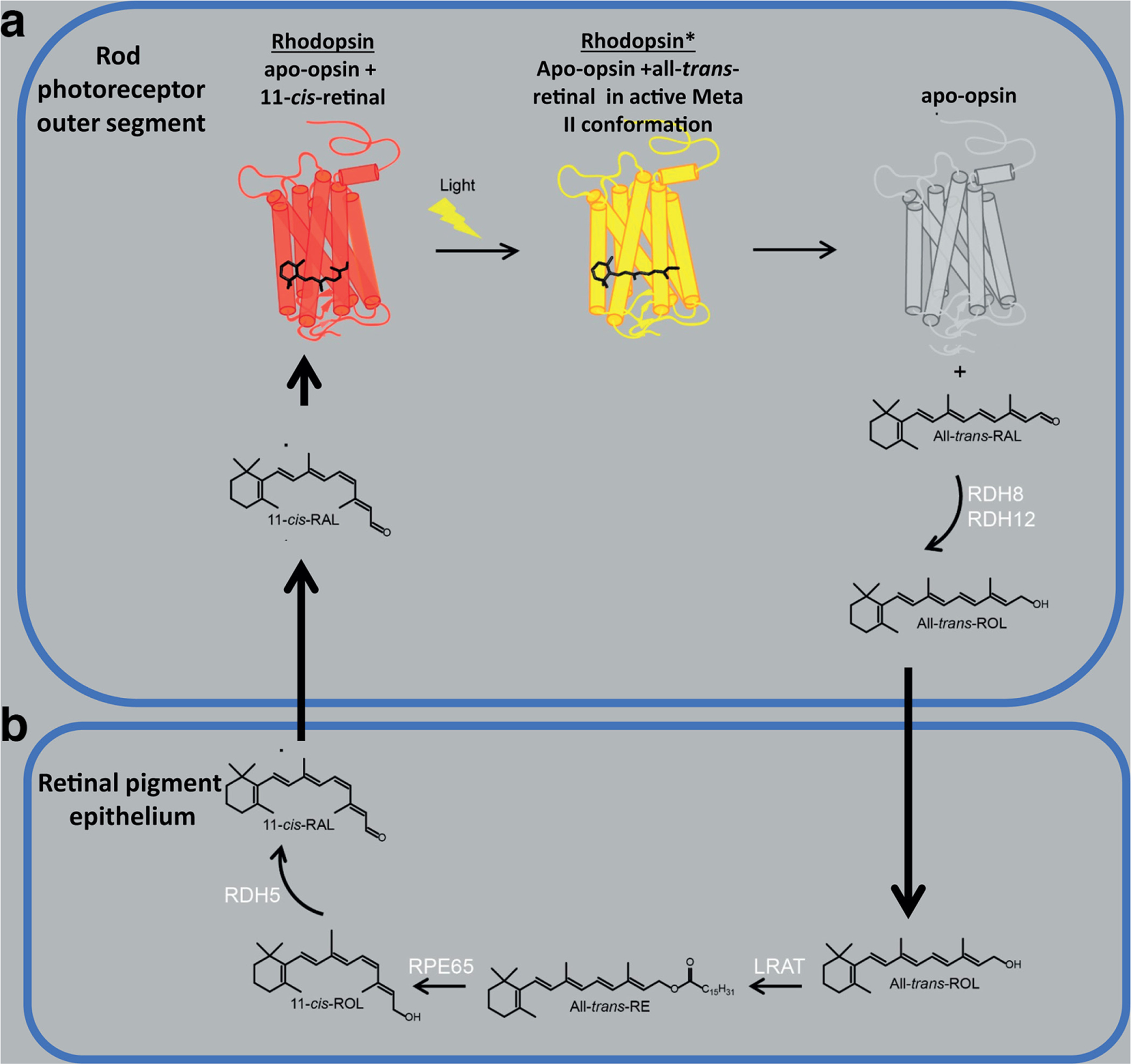
(**a**) 11-*cis*-retinal is associated with rhodopsin in rod outer segments in the dark. When exposed to light, this retinoid undergoes stereoisomerization to all *trans*-retinal to form the Meta II form of the protein. Eventually, all-*trans*-retinal is released from rhodopsin and processed to all-*trans*-retinol. (**b**) All-*trans*-retinol is transferred to the RPE, where regeneration of 11-*cis*-retinal occurs. Abbreviations of retinoid compounds: All-*trans*-RAL, all-*trans*-retinal; All-*trans*-ROL, all-*trans*-retinol; All-trans-RE, All-*trans*-retinylester; 11-*cis*-ROL, 11-*cis*-retinol; 11-*cis*-RAL, 11-*cis*-retinal. Abbreviations of enzymes involved in retinoid synthesis: RDH8, retinal dehydrogenase 8; RDH12, retinal dehydrogenase 12; LRAT, lecithin retinol acyltransferase; RPE65, retinyl pigment epithelium-specific protein 65; RDH5, retinal dehydrogenase 5. Adapted from Ortega and Jastrzebska (2019) Int. J. Mol. Sci. **20,** 6218.

**Figure 4.**
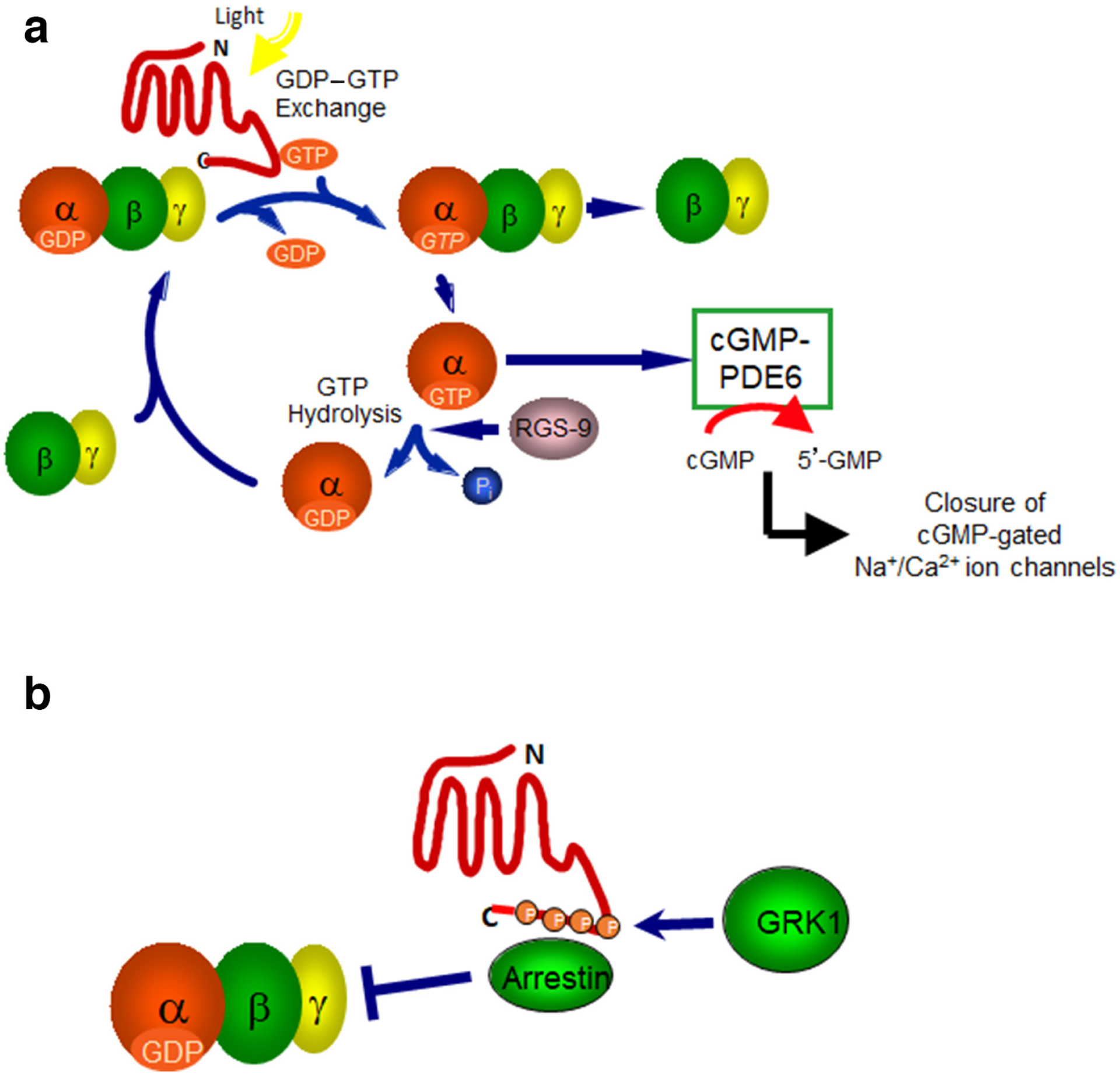
(**a**) Activation of rhodopsin by light stimulates transducin (G_t1_) activation via guanine nucleotide exchange. The GTP-bound α subunit activates phosphodiesterase 6 (PDE6), which hydrolyses cGMP to 5′-GMP, thereby reducing cGMP levels and leading to the closure of the cGMP-gated Na^+^/Ca^2+^ ion channels. Inactivation of Gα_t1_-GTP occurs through hydrolysis of GTP to GDP promoted by the protein, Regulator of G protein Signalling 9 (RGS9). Gα_t_ reassociates with βγ to form the inactive heterotrimeric G protein. (**b**) Inhibition of G protein signalling occurs through phosphorylation of the carboxyl terminus of rhodopsin by GRK1, followed by the binding of arrestin. Arrestin sterically blocks the binding of G_t_.

Rhodopsin is expressed in rod photoreceptors, and three cone opsins [blue, green and red, or more accurately, short-wavelength (SWL) middle-wavelength (MWL) and long-wavelength (LWL) opsins] are expressed in distinct cone photoreceptors in humans and old-world monkeys. Other vertebrates contain different numbers of cone types expressing a variety of cone opsins differing in spectral sensitivity. Rhodopsin and the cone opsins are classical G protein-coupled receptors (GPCRs) that activate the G proteins G_t1_ and G_t2_, respectively. In the human retina, approximately 10^8^ rhodopsins are located in the densely packed discs inside each rod outer segment (Figure 2). In contrast, cones contain disc-like structures that are an extension of the cone outer segment cell membrane (not shown). Sensitivity to light is determined by the high density of the opsins. Light detection by these GPCRs is due to the covalent binding of a derivative of vitamin A, 11-*cis*-retinal (the chromophore), to the apo-opsin proteins (Figure 3a). The specific amino acid sequence of each opsin coupled to 11-*cis*-retinal determines its spectral sensitivity. In rods, for example, upon exposure to light, 11-*cis*-retinal in rhodopsin isomerizes to all-*trans*-retinal. Rhodopsin goes through several rapid conformational changes to generate the active form, metarhodopsin II (Meta II) (Figure 3a). The Meta II forms of the opsins in rods and cones activate their G proteins, which stimulate cGMP hydrolysis by phosphodiesterase 6 (PDE6). This reduction in cGMP causes closure of cGMP-gated, inwardly rectifying Na^+^/Ca^2+^ channels and hyperpolarization of the photoreceptor cell membrane (Figure 4a). A number of complex downstream neuronal pathways integrate the photoreceptor signals via bipolar, horizontal and amacrine cells, which converge on the ganglion cells, resulting in signal transmission from the optic nerve to the brain. It is important to note that light activation causes graded changes in membrane potential, based on the number of opsins that are activated. Each opsin can be activated by a single photon. The more opsins that are activated, the greater the hyperpolarization and the larger the signal transmitted from the photoreceptor to the bipolar cell. Therefore, brighter light results in a larger signal than dim light.

When you close your eyes, the scene before you disappears in approximately 200 mseconds. If you turn around and open your eyes, your vision appears to be restored instantaneously and displays a new scene. This demonstrates that rapid signal turnoff and recovery of the light-exposed opsins to their dark state is required for continuously changing visual perception. The mechanism of signal turnoff also follows a classical GPCR signalling pathway (see Figure 4b for rod pathway). In rods, several serines and threonines at the carboxyl terminus in light-activated rhodopsin (Meta II) are phosphorylated by GRK1, a member of the G protein-coupled receptor kinase (GRK) family. In cones of some species (such as mice and rats), the opsins are also phosphorylated by GRK1. However, in other species, they may be phosphorylated by GRK7 or by both kinases. Phosphorylation leads to the binding of rod or cone arrestins, which sterically block the binding of the G protein, resulting in signal turnoff. Meta II ultimately decays to apo-opsin by releasing all-*trans*-retinal and is no longer active (Figure 3a).

The ability of an opsin to recover light sensitivity under conditions where a moderate number of opsins are bleached is reported to occur in the following order: the release of all-*trans*-retinal and the binding of 11-*cis*-retinal to form dark-adapted rhodopsin. This is followed by the release of arrestin and dephosphorylation of rhodopsin. The 11-*cis*-retinal is provided by the RPE for rods (Figure 3b). For regeneration of cone opsins, a retinoid precursor (11-*cis*-retinol) is provided by Müller glia to cones for the final synthesis of 11-*cis*-retinal (not shown). Reassociation of 11-*cis*-retinal with apo-opsins returns them to the dark, inactive state (known as the ground state) ready to be activated by light (Figure 3a). Guanylyl cyclase is stimulated in the dark to produce more cGMP, resulting in the reopening of cGMP-gated Na^+^/Ca^2+^ ion channels and causing the photoreceptor to become more depolarized, thereby increasing its sensitivity to light.

## Dark adaptation

One of the fascinating aspects of visual function is how the neural retina is able to maintain high sensitivity over such a broad range of light intensities. For example, when we go from a room where we are exposed to bright light to a dimly lit room where we cannot see at all, it takes some time for our eyes to adjust to this darker environment. In fact, the brighter the light in the first room, the longer it takes to adjust our vision in the darker room. This is known as dark adaptation. It has been reported that dark adaptation of rods after a light exposure that activates (bleaches) 90% of the rhodopsin occurs within 30–40 minutes in humans and 1–3 hours in mice. Based on psychophysical experiments, visual sensitivity decreases approximately 100,000-fold in response to a light that bleaches greater than 90% of rhodopsin. Recovery occurs in the dark with a biphasic curve due to the adaptation of cones within the first few minutes, which helps recover some sensitivity, followed by adaptation of rods, which recover more slowly, but result in much greater light sensitivity. The steps that lead to a fully dark-adapted retina capable of light detection (the ground state) requires the binding of new 11-*cis*-retinal to apo-opsin.

Mice have been used extensively to define the mechanism of dark adaptation in rods. However, there is some ambiguity in the order of the steps leading to recovery of rhodopsin bound to its chromophore, 11-*cis*-retinal. This may be due to different experimental conditions in different laboratories, including the intensity and time of light exposure as well as the methods employed for analysing dark adaptation. For example, it has been shown that arrestin binding inhibits dephosphorylation of rhodopsin, indicating that all-*trans*-retinal must be released first, followed by 11-*cis*-retinal binding to allow arrestin to be released and dephosphorylation to occur. Other studies have shown that deletion of an enzyme in the RPE critical for the synthesis of 11-*cis*-retinal has no effect on dephosphorylation of rhodopsin, suggesting the two processes are independent, such that the binding of 11-*cis*-retinal to rhodopsin is not required for rhodopsin dephosphorylation. Alternatively, it has been reported that there is an accelerated rate of rhodopsin decay to apo-opsin plus all-*trans*-retinal in GRK1-knockout mice where rhodopsin is not phosphorylated, suggesting that dephosphorylation of rhodopsin is essential for the efficient decay of Meta II.

To examine the dephosphorylation of rhodopsin during dark adaptation, a mouse model was created in which the catalytic subunit of phosphatase 2A (PP2Acα) was selectively deleted in rods. After a light exposure that bleached approximately 90% of rhodopsin, dark adaptation was markedly delayed due to a severe reduction in the rate of dephosphorylation. However, rhodopsin was eventually dephosphorylated in the absence of PP2Acα, suggesting that a different phosphatase can function in its place, although less effectively. Examination of the retinoid cycle in these PP2Acα-knockout mice during dark adaptation demonstrated a delay in the release of all-*trans*-retinal from rhodopsin and its conversion to all-*trans*-retinol. This resulted in delayed transport of all-*trans*-retinol to the RPE, as well as its conversion to 11-*cis*-retinal and transport back to rods (Figure 3). The data strongly suggest that dephosphorylation of rhodopsin precedes release of all-*trans*-retinal and the subsequent steps that lead to the binding of new 11-*cis*-retinal to rhodopsin. Therefore, dephosphorylation is essential for dark adaptation.

When the PP2A catalytic subunit was selectively deleted in cones, investigators also observed a delay in dark adaptation. Dark adaptation in cones occurs more rapidly, possibly due to the distinct retinoid cycle for cones located in Müller glia rather than in the RPE and the more rapid decay of the cone opsins to apo-opsins. For example, it has been shown that Meta II decays 50 times more rapidly in cones. Nevertheless, dark adaptation of rods and that of cones appear to use fundamentally similar pathways in which dephosphorylation leads to the release of all-*trans*-retinal followed by the synthesis and rebinding of 11-*cis*-retinal to the apo-opsins as the final step in dark adaptation.

## A novel role for cAMP in dark adaptation

Another aspect of regulating dark adaptation may be cAMP dependent. Cyclic AMP levels are high in the dark and low in the light in vertebrate rods. This occurs through a direct effect of light and not through a GPCR signalling pathway. Rods express Ca^2+^/calmodulin-sensitive adenylyl cyclase Type I (AC1). As described above, in the dark, when cGMP-gated ion channels are open, the influx of Ca^2+^ activates AC1 via calmodulin, resulting in higher cAMP levels. When exposed to light, the channels close, reducing AC1 activity. Therefore, cAMP levels are reduced in the light. Work over the last two decades has shown that GRK1 and GRK7 are phosphorylated at Ser21 and Ser23, respectively, in a cAMP-dependent manner in the dark, presumably by cAMP-dependent protein linase (PKA). Based on studies performed *in vitro*, the functional consequence of GRK1 phosphorylation by PKA is to reduce its activity, resulting in a lower rate of rhodopsin phosphorylation. Therefore, phosphorylated GRK1 should be less efficient at phosphorylating rhodopsin. To test this hypothesis *in vitro*, alanine was substituted in place of serine at position 21 within the GRK1 protein, the target site for cAMP-dependent phosphorylation. As expected, this mutation did not allow phosphorylation of GRK1 by PKA. As a consequence, an increased rate of rhodopsin phosphorylation was observed compared to the rate of phosphorylation using wild-type GRK1 phosphorylated by PKA, indicating that the unphosphorylated GRK1 mutant is more active than phosphorylated wild-type GRK1. Therefore, the level of GRK1 phosphorylation is expected to influence the rate of dark adaptation *in vivo* by altering the rate of rhodopsin phosphorylation.

The GRK1 mutant (GRK1-S21A) was substituted for wild-type GRK1 in mice for *in vivo* studies. This mutant had no effect on the response of dark-adapted retinas to a dim flash of light, possibly due to the relative overabundance of this GRK1 mutant compared to the amount of photoactivated rhodopsin. However, as predicted, the rate of dark adaptation was significantly slowed in the mutant mice after a blinding bleach (>90% of rhodopsin), presumably by increasing the rate of rhodopsin phosphorylation. From these studies, we propose that in wild-type mice, there is a progressive reduction in the rate of rhodopsin phosphorylation as cAMP-dependent phosphorylation of GRK1 increases over time in the dark, reducing its kinase activity. Simultaneously, PP2A would be dephosphorylating rhodopsin. The relative rates of rhodopsin phosphorylation by GRK1 and dephosphorylation by PP2A would control the rate of rhodopsin decay to apo-opsin and all-*trans*-retinal and, therefore, the rate of 11-*cis*-retinal synthesis and its ultimate rebinding to apo-opsin to generate ground-state rhodopsin.

Surprisingly, although selective deletion of the catalytic subunit of PP2A in cones also delays dark adaptation, replacing wild-type GRK1 in cones with the GRK1-S21A mutant had no effect. Therefore, a fundamental difference appears to exist between the role of cAMP in dark adaptation in rods and cones, but the reason for this difference is not understood. It should be noted that mouse cones express only GRK1. It may be that cAMP-dependent phosphorylation of GRK7, which is observed in other vertebrates, plays a more important role in vertebrate cones expressing both kinases, such as humans, monkeys and fish, since the deletion of GRK7 slows dark adaptation in zebrafish cones. The importance of these observations for dark adaptation requires further study.

## Conclusions

This review has described several factors that are essential for controlling the rate of dark adaptation, including the dephosphorylation of rhodopsin by PP2A, the release of all-*trans*-retinal and the synthesis of 11-*cis*-retinal followed by its binding to apo-opsin to form the dark-adapted ground state. Whether the binding of 11-*cis*-retinal occurs before dephosphorylation of rhodopsin or after may depend on different experimental conditions such as the intensity of light and the total amount of rhodopsin that is bleached. The timing of arrestin binding and release and its potential role in dark adaptation remain unclear. However, the most recent reports on dark adaptation after a 90% bleach demonstrate that dephosphorylation of rhodopsin by PP2A occurs prior to the release of all-*trans*-retinal. In addition, there may be a role for the regulation of GRK1 activity by cAMP in the dark. Further analysis of these events in rod dark adaptation and a potential role for GRK7 in cone dark adaptation is the focus of ongoing studies.
